# Interest in Rhinoplasty and Awareness of Postoperative Complications Among Female High School Students in Taif, Saudi Arabia

**DOI:** 10.7759/cureus.56741

**Published:** 2024-03-22

**Authors:** Muhanna A Alhusayni, Abrar A Alzahrani, Fahad M Alhomaidi, Raghad N Alotaibi, Liyan K Abu Rukbah, Ghadi F Alotaibi, Marwan F Alnofaie

**Affiliations:** 1 College of Medicine, Taif University, Taif, SAU; 2 Otorhinolaryngology, King Faisal Medical Complex, Taif, SAU

**Keywords:** saudi arabia, taif, postoperative complications, interest, awareness, rhinoplasty

## Abstract

Background and objective

There has been a significant increase in rhinoplasty procedures in Saudi Arabia recently. Cultural factors, societal pressure, and a desire for beauty and self-improvement contribute to the growing interest in cosmetic procedures among the Saudi population. However, limited research has been conducted specifically focusing on the interest in rhinoplasty and the awareness of postoperative complications among female high school students.

This study aimed to investigate the interest in rhinoplasty and awareness of postoperative complications among female high school students in Taif, Saudi Arabia.

Methodology

A cross-sectional observational study was conducted among female high school students through January and February 2024 using a validated questionnaire comprising sociodemographic characteristics, attitudes toward rhinoplasty, and the females’ familiarity with the postoperative complications of rhinoplasty.

Results

The study was conducted among 737 female high school students. About half of the females (*N* = 376, 51%) were 18 years old, and the other half were under 18 years old (*N* = 361, 49%). Out of the total females, only 19 (2.6%) underwent cosmetic surgery, with 6 (60%) opting for rhinoplasty. The study found that 152 (20.6%) females were interested in rhinoplasty, and about a quarter of them (N = 99, 13.4%) did not feel happy about their noses. The most common rhinoplasty complications reported by the respondents were a mismatch of the new nose with the rest of the face (*N* = 471, 63.9%), headache (*N *= 459, 62.3%), breathing disorders (*N *= 458, 62.1%), dissatisfaction with the new nose (*N *= 437, 59.3%), and nose blockage (*N *= 427, 57.9%). It was found that interest in rhinoplasty was significantly more common among participants having relatives or friends who underwent rhinoplasty than others (*N *= 51, 25.8% vs. *N *= 85, 17.2%, respectively; *P *= 0.010). Moreover, it was revealed that interest in rhinoplasty was significantly more common among participants who were not happy with their nose (*N *= 78, 78.8%) than those who were happy with their nose (*N *= 41, 8.3%) and those who did not care (*N *= 33, 22.9%) (*P *< 0.001).

Conclusions

The study revealed that a significant percentage of participants expressed interest in rhinoplasty, indicating a desire for nose reshaping. Counseling services should be available to support students in developing positive body image and self-acceptance. Furthermore, it is important to establish guidelines for media and advertising to guarantee accurate information from surgeons and psychiatrists, ensuring that the public receives balanced and reliable information.

## Introduction

Cosmetic plastic surgery has become increasingly popular in recent years, particularly among young people. In 1999, board-certified plastic surgeons in the United States performed around 600,000 plastic surgery procedures, of which approximately 25,000 were performed on teenagers. Statistics indicate that the number of teenagers undergoing cosmetic surgery in the United States doubled between 1994 and 1998 [1]. According to recent statistics in American society, the number of cosmetic surgeries, such as facelifts and body lifts, as well as nonsurgical treatments like injections and peels, almost tripled between 1997 and 2003 [2]. According to the statistics of 2019, rhinoplasty made up 60% of all plastic surgeries that were performed in Saudi Arabia [3]. Rhinoplasty is one of the most common plastic surgeries, which is a procedure that reshapes the nose to enhance facial appearance [4]. Individuals dissatisfied with the appearance of their nose may choose to undergo rhinoplasty, which alters the shape or appearance of the nose while preserving or improving the nasal airway [5].

The nose is a noticeable face aspect and is often considered a significant beauty feature. Facial appearance is closely associated with self-image, self-confidence, and self-worth. Any irregularities in the nasal structure, whether caused by surgery, trauma, or natural causes, can profoundly impact an individual's emotional well-being [6]. In Saudi Arabia, there has been a significant rise in the number of rhinoplasty procedures conducted in recent years. At present, such operations make up 30% of all cosmetic surgeries performed in the country. While some individuals seek rhinoplasty to correct a dysmorphic facial feature, the influence of beauty standards as portrayed on social media is increasingly motivating people to opt for this surgery [7].

However, rhinoplasty carries various risks and complications due to mismatched expectations between patients and surgeons. Reduction rhinoplasty can lead to breathing difficulties in 70% of revision rhinoplasty patients, while scars and loss of mucosal sensation can also cause a blocked nose sensation. Autogenous transplants of the cartilage (columella, spreader grafts) may dislocate or resorb, while alloplasts can result in infection and extrusion, with silicone implants having a complication rate of 5-20%. Skin and soft tissue complications include atrophy, fibrosis, numbness, cysts, and granulomas [8]. Infections are rare but can be life-threatening, particularly when combined with sinus surgery. Rarely osteotomies can cause orbital injuries, necrosis of eyelids, and rare blindness. Permanent anosmia, damage to the infraorbital nerve, and enophthalmos developing months are also rare complications for rhinoplasty. Other risks were common, including rhinoliquorrhea, brain damage, fistulas, aneurysms, and thrombosis. In addition, dental discoloration and legal disputes are also possible. Postoperative deformities, such as the Polly beak, broad nasal tip, columellar retractions, and dorsal irregularities, are common and often require revision surgery. Skill and experience can reduce complications, but ongoing education and distinguishing between complications and mistakes are crucial [8]. According to the literature, the complication rate for rhinoplasty procedures is between 4% and 18.8%. In life-threatening cases, 1.7% to 5% of complications occur [9]. Studies have shown that young adults, particularly teenage females, have insufficient knowledge about the procedure and its potential complications in Iran [10] and in Riyadh, Saudi Arabia, among females aged from 14 to 17 years [11].

Exploring the reasons behind the interest in rhinoplasty among female high school students can help us understand the impact of cultural and societal norms on their perception of beauty and their desire for cosmetic procedures. It is crucial to assess the awareness of postoperative risks and complications associated with rhinoplasty among high school students to gain insights into their understanding of the potential dangers of such surgeries. This information can be used to develop educational programs and interventions aimed at improving their knowledge about the risks involved in cosmetic surgeries. Therefore, our study aims to investigate the interest in rhinoplasty and awareness of postoperative complications among female high school students in Taif, Saudi Arabia.

## Materials and methods

Study design and duration

This cross-sectional study was performed among female high school students in Taif, Saudi Arabia. Data were collected through January and February 2024.

Study population

The study included female high school students aged 16 to 18 years old living in Taif, who were willing to participate in the study. Males were excluded from the study.

Sample size calculation

The Raosoft sample size calculator was used online to calculate the sample size. Based on a confidence level of 99%, a margin of error of 5%, and a maximum uncertainty of 50% for positive responses, a minimum of 643 participants should be included in this study.

Data collection

Data were collected through a structured and self-administered electronic questionnaire distributed to female high school students in Taif. The questionnaire was validated and used in a previous study conducted in Iran [10]. The data collection tool consists of three sections. The first section included the participants' characteristics such as age, number of family members, their parents’ education and income status, and medical insurance. The second section included questions about attitudes toward rhinoplasty, whereas the third section included questions about the students’ familiarity with the postoperative complications of rhinoplasty.

Data entry and analysis

Data were extracted and revised in an Excel sheet. Statistical analysis was conducted using the IBM SPSS computer program (version 26.0, IBM Corp., Armonk, NY). Categorical variables were expressed in numbers and percentages. The chi-square test and Fisher’s exact test were used to compare between different categorical variables. The statistical significance was established by considering *P*-values < 0.05.

Ethical considerations

Approval was given by the research and ethical committee of the Scientific Research Ethics Committee at King Faisal Medical Complex in Taif (Approval number: 2024-E-3). In addition, the study’s objective was illustrated at the beginning of the questionnaire.

## Results

The study included 737 female students in the high school. About half of them (*N* = 376, 51%) were 18 years old and had family members more than six members (*N *= 385, 52.6%). Regarding their parents' education, more than half of the fathers had a high level of education, with 105 (14.2%) holding a Master's or Doctorate. Among mothers, 331 (44.9%) had a bachelor's degree, and 54 (7.3%) held a Master's or Doctorate. Regarding medical insurance, 302 (41%) had government insurance, and 109 (14.8%) had private insurance. About half of the participants (*N* = 374, 50.7%) had families with incomes ranging from 10,000 to 20,000 SAR. A small percentage (*N *= 19, 2.6%) had undergone cosmetic surgery before, whereas 6 (60%) had undergone rhinoplasty. About one-third of them (*N *= 198, 28.6%) had relatives or friends who had undergone a rhinoplasty, as shown in Table 1.

**Table 1:** Demographic characteristics of the participants (N = 737). Data are presented as numbers and percentages.

Parameters	Category	N	%
Age (Years)	16	125	17.0
	17	236	32.0
	18	376	51.0
Number of family members (*N* = 732)	1-6	347	47.4
	>6	385	52.6
Father’s education	Under high school	77	10.4
	High school	264	35.8
	Bachelor degree	291	39.5
	Master’s or Doctorate	105	14.2
Mother’s education	Under high school	147	19.9
	High school	205	27.8
	Bachelor degree	331	44.9
	Master’s or Doctorate	54	7.3
Income of family	<10,000 SAR	169	22.9
	From 10,000-20,000 SAR	374	50.7
	>20,000 SAR	194	26.3
Medical insurance	Government	302	41.0
	Private	109	14.8
	None	326	44.2
Have you ever had any cosmetic surgery?	Yes	19	2.6
	No	718	97.4
If you have had plastic surgery, what is it? (*N* = 10)	Rhinoplasty	6	60
	Unwinding process	2	20
	Mole removal	1	10
	Beautification of the eyebrow due to a deep aperture	1	10
Number of relatives or friends who have had a rhinoplasty (*N* = 737)	None	495	71.4
	One	110	15.9
	Two	69	10.0
	Three	19	2.7

**Figure 1:**
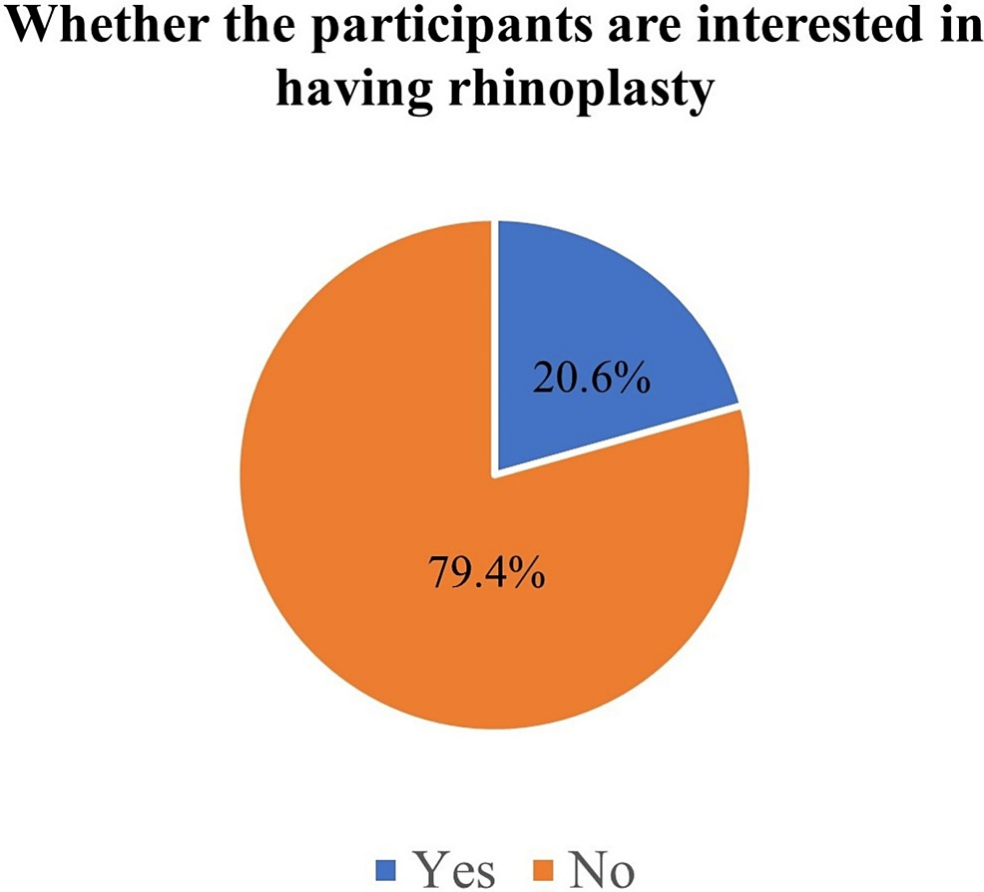
Whether the participants are interested in having rhinoplasty. Data are presented in the figure as percentages.

The number of females interested in rhinoplasty was 152 (20.6%), as shown in Figure 1.

Regarding the attitude of females toward rhinoplasty, about a quarter of them (*N *= 99, 13.4%) did not feel happy about their noses, and 152 (20.6%) wanted to undergo rhinoplasty. About half of them (*N *= 304, 56.1%) wished to undergo it for beauty. The majority of them (*N* = 481, 82.6%) indicated they preferred a doctor who performed well to operate on them. About half of those who did not want to undergo rhinoplasty (*N* = 374, 54.8%) reported that their reason was preferring their nose shape.

Regarding the knowledge about rhinoplasty complications, only 241 (32.7%) had information about skin discoloration, and 369 (50.1%) knew about recurrent nosebleeds. In addition, 427 (57.9%) knew about nose blockage, and 265 (36%) knew about recurrent nasal mucosal irritation. Furthermore, 459 (62.3%) had information about headaches, 226 (30.7% ) knew about recurrent nausea and vomiting, and 345 (46.8%) knew about nasal discharge, which could occur as an early complication. While 458 (62.1%) knew about breathing disorders, 394 (53.5%) were aware of sensitivity to strong odors. Approximately two-thirds had information about mismatching the new nose with the rest of the face (*N* = 471, 63.9%), 437 (59.3%) knew about dissatisfaction with the new nose, and 329 (44.6%) were aware of the need for reoperation. However, only 126 (17.1%) were aware of the possibility of death, which could occur as a late complication. All the details are shown in Table 2.

**Table 2:** Attitude of females toward rhinoplasty and their knowledge about its complications. Data are presented as numbers and percentages.

Parameters	Category	N	%
Attitude			
How do you feel about your nose?	Happy	494	67.0
	Not happy	99	13.4
	Do not care	144	19.5
Do you want to do a rhinoplasty?	Yes	152	20.6
	No	585	79.4
Why do you want to do a rhinoplasty? (*N* = 542)	For beauty	304	56.1
	Because of the insistence of friends and family	7	1.3
	To show off	7	1.3
	To catch up with the mode	14	2.6
	Others	210	38.7
What type of doctor would you like to operate on you? (*N* = 582)	The doctor who works well	481	82.6
	Doctor with good manners	21	3.6
	The doctor who charges less	13	2.2
	The doctor who has lots of patients	22	3.8
	Others	45	7.7
Why do you not want a rhinoplasty? (*N* = 683)	I like my nose as it is	374	54.8
	Do not have the financial capacity	36	5.3
	Plastic surgery is of no use	35	5.1
	Fear of operation	72	10.5
	Fear of side effects	91	13.3
	Others	75	11.0
Knowledge about rhinoplasty complication			
Skin discoloration	Yes	241	32.7
	No	496	67.3
Breathing disorders	Yes	458	62.1
	No	279	37.9
Recurrent nosebleed	Yes	369	50.1
	No	368	49.9
Nose blockage	Yes	427	57.9
	No	310	42.1
Recurrent nasal mucosal irritation	Yes	265	36.0
	No	472	64.0
Headache	Yes	459	62.3
	No	278	37.7
Recurrent nausea and vomiting	Yes	226	30.7
	No	511	69.3
Nasal discharge	Yes	345	46.8
	No	392	53.2
Sensitivity to strong odors	Yes	394	53.5
	No	343	46.5
Death	Yes	126	17.1
	No	611	82.9
Need for reoperation	Yes	329	44.6
	No	408	55.4
Dissatisfaction with the new nose	Yes	437	59.3
	No	300	40.7
Mismatch of the new nose with the rest of the face	Yes	471	63.9
	No	266	36.1

Table 3 shows the correlation between the interest in rhinoplasty and sociodemographic characteristics. It was found that interest in rhinoplasty was significantly more common among participants having relatives or friends who underwent rhinoplasty than others (*N* = 51, 25.8%, vs. *N* =85, 17.2%, respectively; *P *= 0.010). Moreover, it was revealed that interest in rhinoplasty was significantly more common among participants who were not happy with their nose (*N *= 78, 78.8%) than those who were happy (*N *= 41, 8.3%) and those who did not care (*N *= 33, 22.9%) (*P *< 0.001).

**Table 3:** Correlation between the interest in rhinoplasty regarding the sociodemographic characteristics. Data are presented as numbers and percentages. The correlation is represented as *P*-values, where a value below 0.05 is statistically significant and displayed in bold. ^*^Fisher’s exact test.

Factors	Interest in rhinoplasty, *N* (%)		*P*-value
	Yes	No	
Age (Years)			
16	27 (21.6)	98 (78.4)	0.956
17	48 (20.3)	188 (79.7)	
18	77 (20.5)	299 (79.5)	
Number of family members			
1-6	64 (18.4)	283 (81.6)	0.233
More than 6	85 (22.1)	300 (77.9)	
Father’s education			
High school or below	65 (19.1)	276 (80.9)	0.331
Bachelor or higher	87 (22.0)	309 (78.0)	
Mother’s education			
High school or below	72 (20.5)	280 (79.5)	0.913
Bachelor or higher	80 (20.8)	305 (79.2)	
Medical insurance			
Government	69 (22.8)	233 (77.2)	0.345
Private	18 (16.5)	91 (83.5)	
None	65 (19.9)	261 (80.1)	
Have you ever had any cosmetic surgery?			
Yes	6 (31.6)	13 (68.4)	0.250*
No	146 (20.3)	572 (79.7)	
Having relatives or friends underwent rhinoplasty			
Yes	51 (25.8)	147 (74.2)	0.010
No	85 (17.2)	410 (82.8)	
Income of family			
<10,000 SAR	41 (24.3)	128 (75.7)	0.222
From 10,000 to 20,000 SAR	68 (18.2)	306 (81.8)	
>20,000 SAR	43 (22.2)	151 (77.8)	
Perceived happiness about nose			
Happy	41 (8.3)	453 (91.7)	<0.001
Not happy	78 (78.8)	21 (21.2)	
Do not care	33 (22.9)	111 (77.1)	

## Discussion

Our research aimed to investigate the interest in rhinoplasty and awareness of postoperative complications among female high school students in Taif.

We found that 152 (20.6%) females were interested in rhinoplasty. Similar studies conducted in Saudi Arabia in different regions found that 25.7% in Riyadh [11] and 22.62% in the Western Region [12] were interested in rhinoplasty, which was consistent with our results. However, another study in Iran [10] among female high school students reported a higher percentage than ours, with 53.65 % of them interested in rhinoplasty [10]. That could contribute to the diversity of cultures. Additionally, parental education has been identified as a mediating factor that may impact the child's interest. A prior study has suggested that as parental education level increases, the rate of cosmetic surgery decreases [13]. However, our study showed no significant association between the level of parental education and interest in rhinoplasty. Another study in Iran showed that interest in rhinoplasty increased with parents' education, but the increase was not significant [10].

Our study found that individuals who were unhappy with the shape of their noses were more likely to be interested in rhinoplasty. This finding was in line with a previous study conducted in Riyadh [11]. However, another study conducted in Iran found no significant correlation between participants' satisfaction with their nose shape and their interest in rhinoplasty [10]. In addition, our study also revealed that having friends or relatives who underwent rhinoplasty was a significant factor in an individual's interest in the procedure. This finding was consistent with the results obtained from a study conducted in Riyadh [11]. Moreover, another study in California reported that 30% of participants were influenced by someone they knew who had undergone rhinoplasty [1].

Regarding the attitude of our participants, about two-thirds of them (*N* = 494, 67%) were satisfied with the appearance of their noses. This finding was consistent with studies conducted in Saudi Arabia, where a study in the Western region [12] found that 62.5% of participants were happy with their noses, and another study in Riyadh reported that 48.7% were satisfied with their noses [11]. Similarly, a study conducted in Iran found that almost half of the participants (46.96%) were happy with their noses, which reflected that 59.18% did not want to undergo rhinoplasty surgery because they liked their nose shape [10]. It aligned with our study, whereas 54.8% of participants who did not want to undergo rhinoplasty reported that they were satisfied with their nose shape, followed by concerns about the side effects of the surgery. This was consistent with the findings of the studies conducted in Riyadh [11] and the Western region of Saudi Arabia [12], where participants reported being happy with their nose as it was, followed by religious beliefs, concerns about side effects, and financial costs.

The most reported reason for doing rhinoplasty is beauty, which aligns with previous studies. An Iranian study found that people were motivated by beauty and staying fashionable [10]. Another study in California [1] revealed that 90% of those opting for cosmetic surgery did so to feel better about themselves. Furthermore, a study in Norway found that young women with body dysmorphic disorder were more likely to be interested in rhinoplasties [14].

 In our research, most female students chose surgeons based on their ability to work well, similar to a study conducted in the western region of Saudi Arabia [12]. Additionally, another study in Iran [10] reported that the majority of participants referred to a doctor who operated well. In contrast, a study in Riyadh [11] indicated that most participants preferred experienced cosmetic surgeons who had performed many successful operations. It was found that a surgeon's knowledge, ability, and proficiency were essential to patients, along with their characteristics [15]. Therefore, selecting a qualified and experienced surgeon can help increase awareness and prevent rhinoplasty complications [16].

The most common rhinoplasty complications reported by females was a mismatch of the new nose with the rest of the face, followed by headache, breathing disorders, dissatisfaction with the new nose, and nose blockage. However, half of them are aware of the need for reoperation, and only a small percentage of females are aware of the possibility of death. These findings are consistent with a study conducted in Riyadh [11], which showed that dissatisfaction with the new nose, headache, and sinus blockage were common complications. Still, more severe complications, such as the need for reoperation and the possibility of death due to infection, were not considered. In Saudi Arabia, the prevalence of reoperations of rhinoplasty was 44.7% [17]. Another study conducted in the western region of Saudi Arabia [12] reported that breathing disorders were the most recognized complication, while headache, nausea, and vomiting were the least recognized. Moreover, in Iran [10], complications recognized by participants were more trivial, such as reoperation, dissatisfaction, and mismatch of the new nose with the rest of the face, and their knowledge of more severe complications, such as headache, nausea and vomiting, and death, was much less. Recently, there has been false advertising of rhinoplasty on social media by influencers who have undergone the procedure, contributing to the general public's lack of awareness of rhinoplasty complications [18,19]. A study reported that nearly 74% of participants believed that rhinoplasty is a safe surgical procedure, highlighting the impact of social media advertising on public perception [20]. Another study found that most participants felt that rhinoplasty is a safe procedure, which supports the hypothesis that the public is unaware of the potential complications associated with the procedure [21]. Therefore, collaborations between medical experts can be established to organize seminars and workshops that provide accurate information and address misconceptions. Media and advertising guidelines should also be developed to provide balanced and accurate information.

Limitations

The validity of our findings is limited as the nature of the study design was a cross-sectional observational study using a self-administered questionnaire that was conducted in a single place in Saudi. That may lead to collecting data at a single point in time. In addition, it is important to note that this study may not have taken into account all potential factors that could impact interest in rhinoplasty. As a result, it is highly recommended that future studies be conducted as generalized studies and a more comprehensive investigation of all possible variables that could affect the interest in rhinoplasty.

## Conclusions

Our research focused on investigating the interest in rhinoplasty and awareness of postoperative complications among female high school students in Taif. The study revealed that a significant percentage of participants expressed interest in rhinoplasty, indicating a desire for nose reshaping. Many females expressed dissatisfaction with their noses, and the influence of friends or family members who had already experienced the procedure contributed to their interest. To address this, educational programs should provide accurate information regarding rhinoplasty's potential risks and outcomes. It is crucial to establish counseling services to foster positive body image and self-acceptance among students. Additionally, guidelines provided by professionals for media and advertising should be developed to ensure that the public receives balanced and accurate information. Patients and their parents should be provided with a document that explains the operation and its associated risks and complications. In certain cases, it may be necessary to consult a psychologist or psychiatrist.
